# Simple oral mucosal epithelial transplantation in a rabbit model

**DOI:** 10.1038/s41598-019-54571-7

**Published:** 2019-12-02

**Authors:** Aya Inamochi, Akiko Tomioka, Kohdai Kitamoto, Takashi Miyai, Tomohiko Usui, Makoto Aihara, Satoru Yamagami

**Affiliations:** 1 Department of Ophthalmology, Graduate School of Medicine, The University of Tokyo, Tokyo, Japan; 2 Division of Ophthalmology, Department of Visual Sciences, Nihon University School of Medicine, Tokyo, Japan; 3 Department of Ophthalmology, Graduate School of Medicine, International University of Health and Welfare, Tokyo, Japan

**Keywords:** Regeneration, Corneal diseases

## Abstract

This study investigated a rabbit model of autologous simple oral mucosal epithelium transplantation (SOMET) for limbal stem cell deficiency (LSCD). LSCD was created in the SOMET group and the Control group. In the SOMET group, oral mucosa harvested from the buccal region was treated with dispase, cut into small pieces, and placed on the exposed corneal stroma without using graft sutures, amniotic membrane, and/or glue. A soft contact lens was positioned and tarsorrhaphy was performed in both groups. Postoperative corneal neovascularization and fluorescein staining scores were evaluated by slit lamp microscopy in both groups. At 2 weeks postoperatively, eyes were excised and subjected to immunohistochemical staining for CK3, CK13, CK15, and p63. In the SOMET group, transplantation of oral mucosa led to complete recovery of LSCD, as indicated by low neovascularization scores, low fluorescein staining scores, and detection of stratified K3/K13-positive cells on the stroma at 2 weeks after surgery. In contrast, corneal epithelial defects persisted in the Control group at 2 weeks. SOMET achieved re-epithelialization of the corneal surface in this rabbit LSCD model. It is a simple technique that does not require culture and could be a promising option for ocular surface reconstruction in bilateral LSCD.

## Introduction

Corneal epithelial stem cells are localized in the basal layer of the corneal limbus^1–3^. When the limbus is damaged by chemical burns, Stevens-Johnson syndrome, autoimmune disease, or other insults, conjunctival invasion of the cornea occurs along with neovascularization and infiltration of fibroblasts, which can impair visual function by reducing corneal smoothness and transparency^4^. This situation arises because of limbal stem cell deficiency (LSCD). While patching with amniotic membrane may achieve recovery of LSCD in some circumstances^5^, an additional source of limbal stem cells (LSCs) is required for long-term maintenance of transparency in patients with complete LSCD^6,7^. Stem cell-based reconstruction of the ocular surface for LSCD has progressed from application of allografts such as conjunctival epithelial grafts^8,9^ and limbal transplantation^10,11^ to use of autografts for avoidance of rejection, including corneal limbal transplantation^12^ and cultured corneal epithelial sheet transplantation^13^ in patients with unilateral LSCD. In addition, bilateral LSCD has been treated by transplantation of cultured conjunctival epithelial cell sheets^14^ and cultured oral mucosal epithelial cell sheets^15–19^. Among these techniques, transplantation of cultured cell sheets reduces the amount of donor tissue required and allows early rehabilitation after surgery. However, this method has certain drawbacks, such as the need for expensive facilities and careful monitoring when manufacturing epithelial cell sheets, as well as legal restrictions.

Sangwan *et al*. developed a tissue transplantation technique called simple limbal epithelial transplantation (SLET)^20^ and reported comparable results to those obtained after transplantation of tissue grafts or cell sheets^21^. The SLET technique has advantages for both patients and the medical system, since cell culture is not required and the risk of causing LSCD in the donor eye is minimized by reducing the donor tissue volume. Recently, allograft SLET was reported in patients who could not undergo autografting^22^. However, considering the risk of allograft rejection, it is worthwhile to develop an autograft-based corneal reconstruction method. We have previously performed simple conjunctival tissue transplantation for bilateral LSCD and confirmed a certain level of efficacy (Yamagami S, personal communication). However, patients with bilateral LSCD often also develop symblepharon, which results in insufficient conjunctival stem cells for reconstruction of the ocular surface. Therefore, methods based on transplantation of other autologous tissues are needed.

Accordingly, we investigated a novel technique for ocular surface reconstruction using uncultured oral mucosal epithelium and evaluated its efficacy in a rabbit model of LSCD.

## Results

### Donor oral mucosa

**Figure 1:**
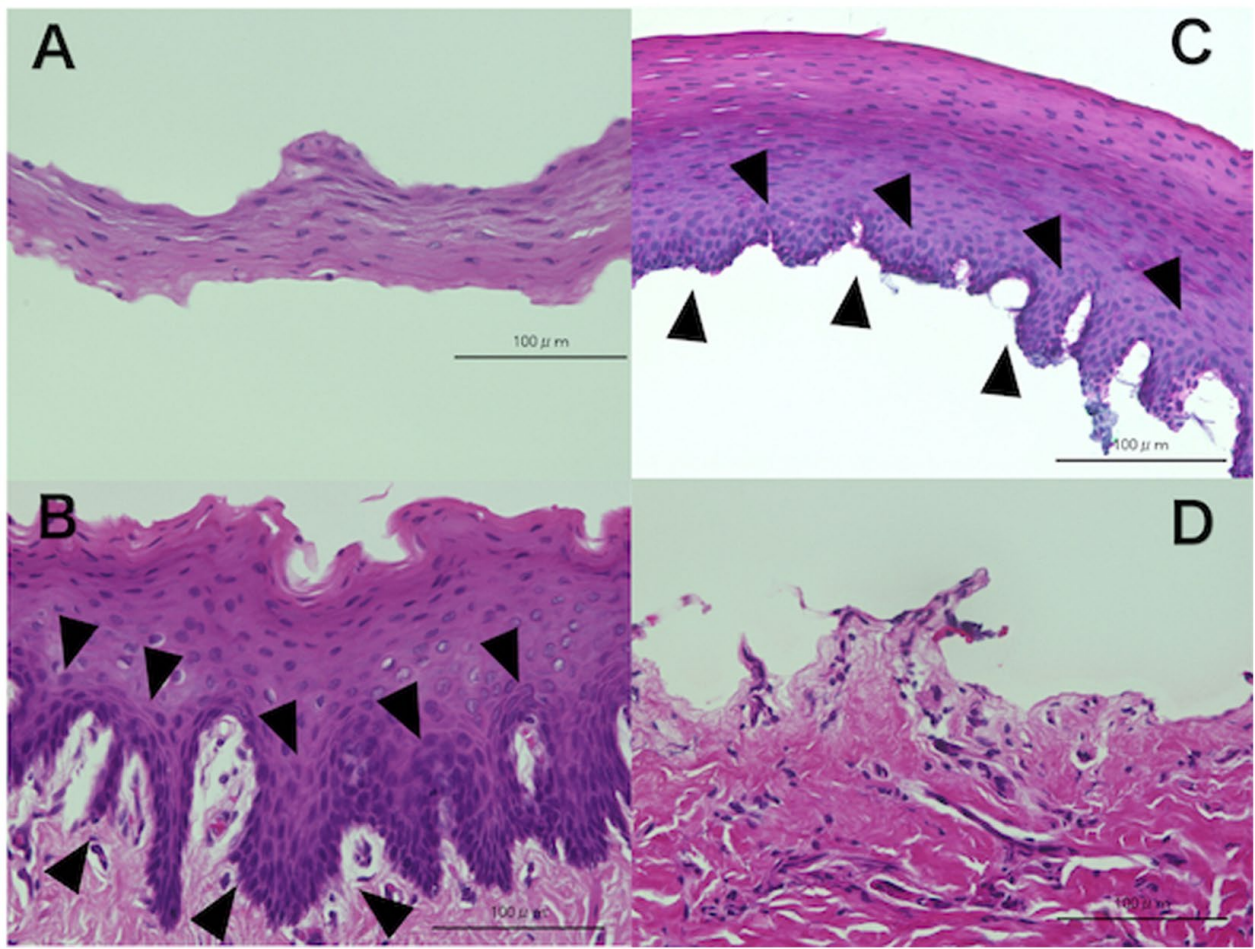
Hematoxylin-eosin staining of harvested rabbit oral mucosal tissue. The surface layer was removed from rabbit oral mucosal tissue with 2 pairs of forceps, dividing the tissue into 2 layers. (**A**) The upper five to six layers of cells in the mucosal epithelium are shown. (**B**) The lower layer of oral mucosa contained basal cells with a high N/C ratio (▲) in the basal layer and near the submucosal connective tissue. (**C**) After treatment with 1.2 IU of Dispase II, submucosal connective tissue was separated from the mucosa. The stratified oral mucosal epithelium contained an irregular basal layer. (**D**) Submucosal tissue and resident mononuclear cells after treatment with 1.2 IU of Dispase II. There are no basal cells in the submucosal connective tissue.

The harvested oral mucosa was composed of mucosal epithelium and subepithelial tissue. Two methods for removal of the subepithelial tissue from the epithelium were tested. (1) Blunt peeling with 2 pairs of forceps separated the tissue into two layers, which were mucosal epithelium (Fig. 1A) and submucosal tissue. Cells with a high nuclear/cytoplasmic (N/C) ratio were identified in the submucosal tissue close to the basal layer, as shown in Fig. 1B. (2) After incubation with dispase (Dispase II, neutral protease, grade II; Roche Diagnostics K.K, Basel, Schweiz), residual submucosal tissue was easily detached from the mucosal epithelium by light application of forceps. The mucosal epithelium thus obtained contained the entire basal layer (Fig. 1C), while no epithelium remained on the submucosal tissue layer (Fig. 1D).

### Culture of separated oral mucosal epithelial layers for immunohistochemical analysis

**Figure 2:**
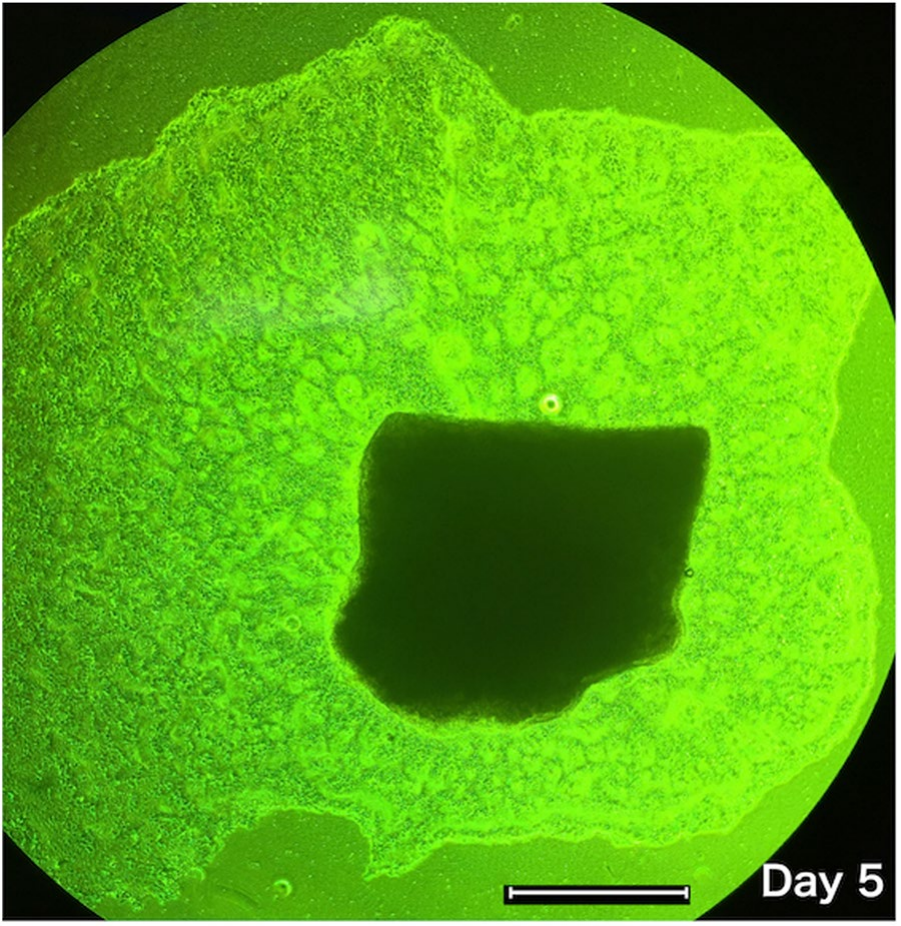
Culture of oral mucosal epithelial cells after dispase treatment. Colony formation was noted around the clumps of cultured cells. Gradual proliferation around the clumps of cells formed round areas of epithelium by day 5 of culture. Scale bar = 1000 µm.

Mucosal epithelium containing the entire basal layer (Fig. 1C), the surface layer of mucosal epithelium (Fig. 1A), and submucosal tissue (Fig. 1D) were cultured separately for 3 weeks. Mucosal epithelium treated with dispase became adherent to the culture dish on day 2 and proliferating cells formed colonies around the cell clumps on day 5 (Fig. 2). In contrast, the surface layer of mucosal epithelium (Fig. 1A) and the submucosal tissue (Fig. 1D) did not become adherent to the culture dishes and cell proliferation was not observed (not shown). These findings suggested that the dispase-treated basal cell layer of mucosal epithelium was suitable for ocular surface reconstruction.

### Postoperative course

**Figure 3:**
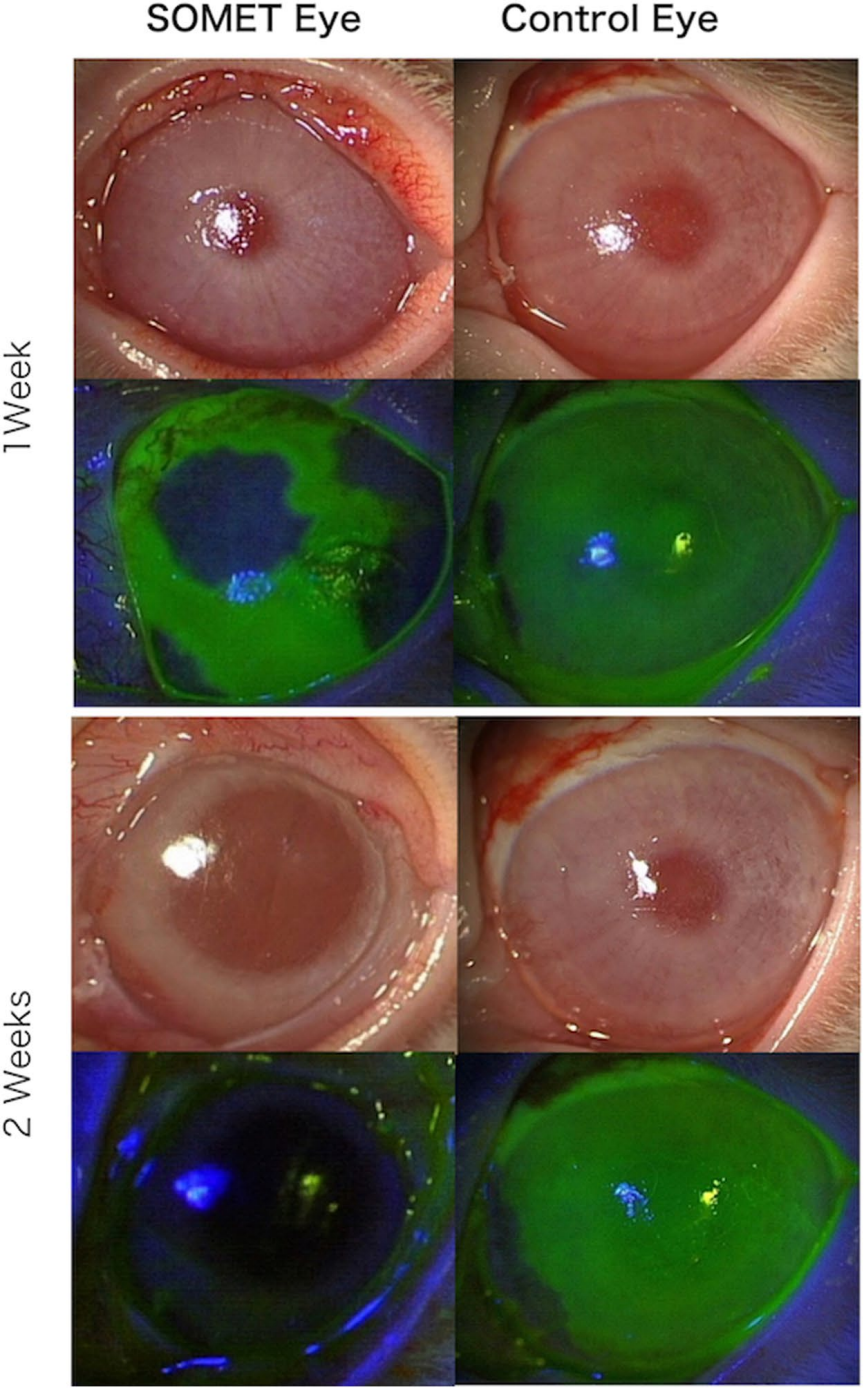
Postoperative slit lamp findings in the SOMET and Control groups. In the SOMET group, epithelium is expanding over the corneal surface around the grafts at 1 week after surgery and epithelialization is complete by 2 weeks. No fluorescein staining of the cornea is observed at 2 weeks (bottom left). In the Control group, neovascularization is present at the periphery of the cornea. Epithelialization of the corneal surface shows no progression and most areas are still fluorescein-positive at 1 and 2 weeks after surgery (bottom right).

**Figure 4:**
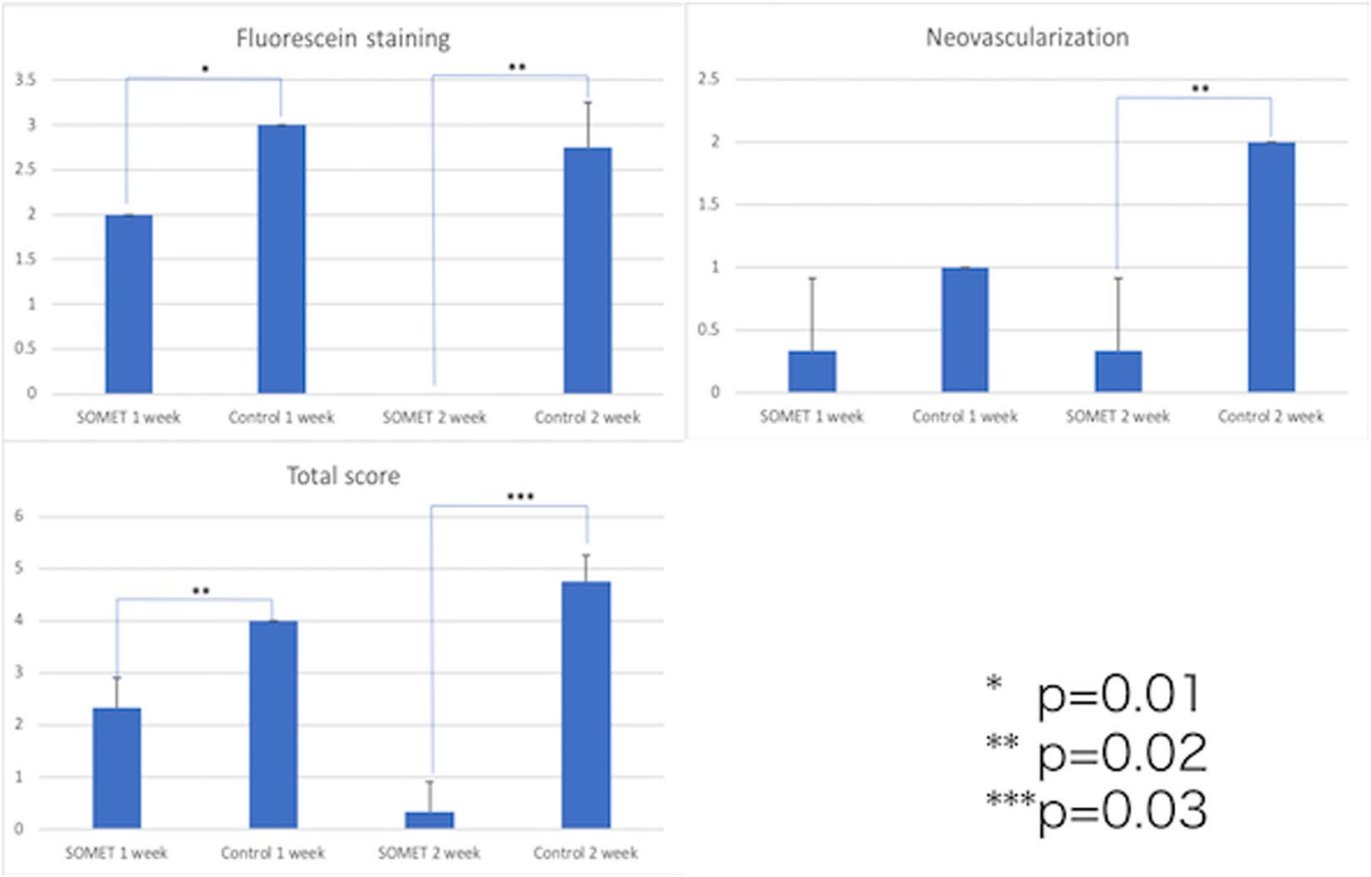
Postoperative clinical scores. Clinical scores were compared between the SOMET group (n = 3) and the Control group (n = 4). At 1 week after surgery, the fluorescein staining score (*p = 0.01) and the total scores (**p = 0.02) were significantly lower in the SOMET group compared with the Control group. At 2 weeks after surgery, the corneal neovascularization score (**p = 0.02), fluorescein staining score (**p = 0,02), and total scores (***p = 0.03) were significantly lower in the SOMET group than in the Control group.

One of the 4 eyes in the SOMET group was excluded from evaluation because the soft contact lens (SCL) dropped off and most of the grafts were lost by the first week after surgery. Figure 3 shows representative anterior segment photos from both groups. In all 3 eyes of the SOMET group, epithelium expanded to form islands around the grafts at 1 week after surgery and the corneal defect was completely epithelialized at 2 weeks. The conjunctiva adjacent to the cornea was stained with fluorescein, but there was no corneal staining, indicating that conjunctival epithelium was not invading the corneal surface (Fig. 3 left). In contrast, neovascularization was severe in the 4 eyes of the Control group at 2 weeks after surgery, unlike the SOMET group. Despite treatment with ointment containing betamethasone sodium phosphate and fradiomycin sulfate, conjunctival inflammation was more prominent in the Control group than in the SOMET group and extensive symblepharon was noted. None of the eyes achieved complete epithelialization within 2 weeks in the Control group (Fig. 3 right). Figure 4 shows the clinical scores obtained after surgery. At 2 weeks postoperatively, neovascularization scores were significantly lower in the SOMET group compared with the Control group (p = 0.02), while fluorescein staining scores were significantly lower in the SOMET group at both 1 week (p = 0.01) and 2 weeks (p = 0.02). Total scores were also significantly lower in the SOMET group than the Control group at 1 week (p = 0.02) and 2 weeks (p = 0.03) postoperatively (Fig. 4).

### Immunohistochemical comparison between *ex vivo* and cultured oral mucosal epithelium

**Figure 5:**
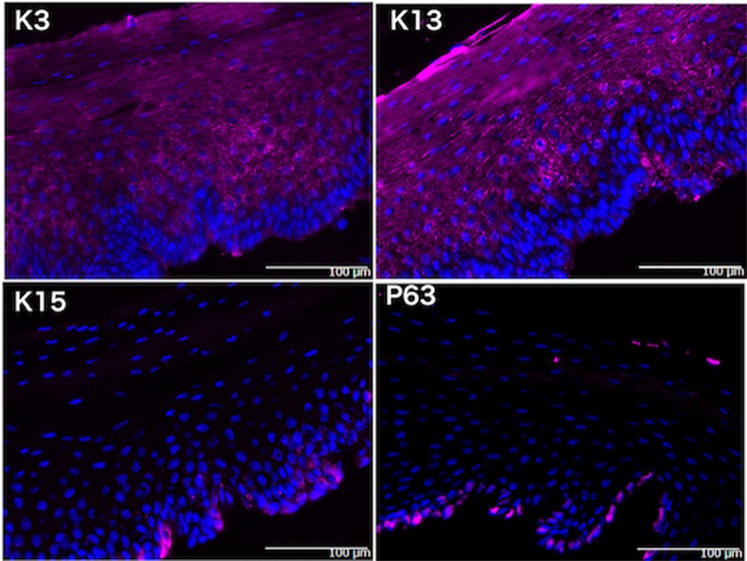
Immunohistochemical staining of oral mucosal epithelium. Submucosal connective tissue was removed by dispase treatment and the superficial tissues were used for immunohistochemistry. Apart from 3 to 5 basal layers, the oral mucosal epithelial cells are diffusely stained by K3 (top left) and K13 (top right), consistent with the phenotype of oral mucosal epithelium. Some basal cells show cytoplasmic positivity for K15 (bottom left), while there is nuclear P63 positivity in the lowest basal cell layer (bottom right).

**Figure 6:**
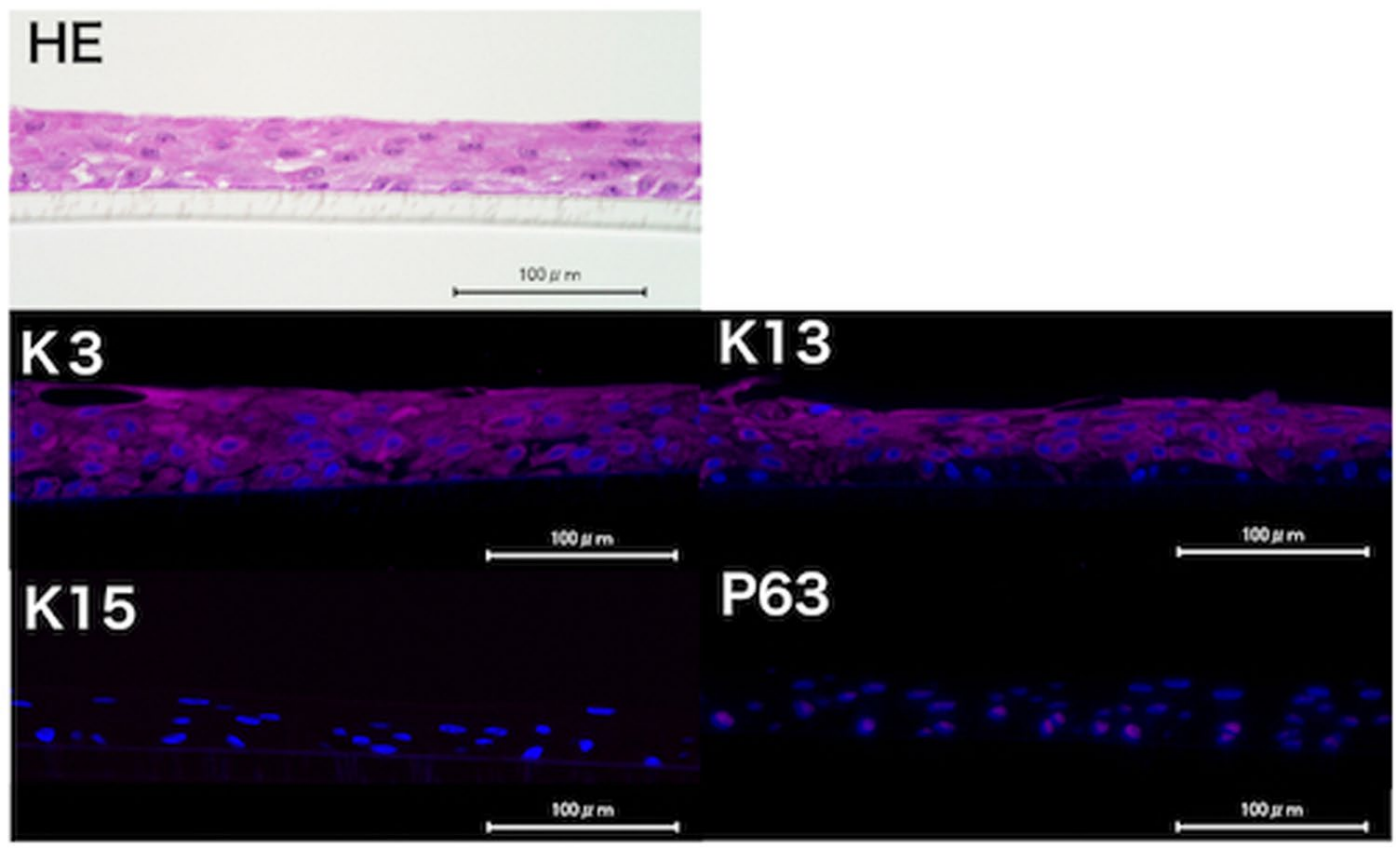
Immunohistochemistry of cultured oral mucosal epithelium. Oral mucosal epithelium was cultured with B-27 and EGF to form epithelial sheets that were subjected to immunohistochemistry. On hematoxylin eosin staining, the cultured oral mucosal epithelial sheets had a smooth five-layer structure. Immunohistochemical staining showed cytoplasmic K3 positivity in all of the cell layers and K13 positivity is all layers except the basal layer. Nuclear positivity for p63 was seen in the basal layer. On the other hand, K15 was completely negative.

After dispase treatment and removal of submucosal connective tissue, the oral mucosal epithelium was subjected to immunohistochemical examination. K3 and K13 were positive in all cell layers, except 3 to 5 layers of the basal cells, while K15 was positive in some basal cells and p63 was positive in the nuclei of most basal cells (Fig. 5). HE staining revealed that the cultured oral mucosal sheets had a smooth five-layer structure (Fig. 6, top left). Immunohistochemical staining showed cytoplasmic K3 positivity in all of the cell layers (middle left), and all cell layers were also K13-positive except for the basal layer (top right). Nuclear positivity for p63 (bottom right) was found in some cells of the basal layer, but there were no K15-positive cells (bottom left). These findings suggested that the phenotype of the cultured cells was consistent with that of oral mucosal epithelium and that most undifferentiated cells disappeared during culture in medium containing B27 (Thermo Fisher Scientific, Waltham, MA) and epidermal growth factor (EGF 10 ng/ml; Peprotech, Rocky Hill, NJ).

**Figure 7:**
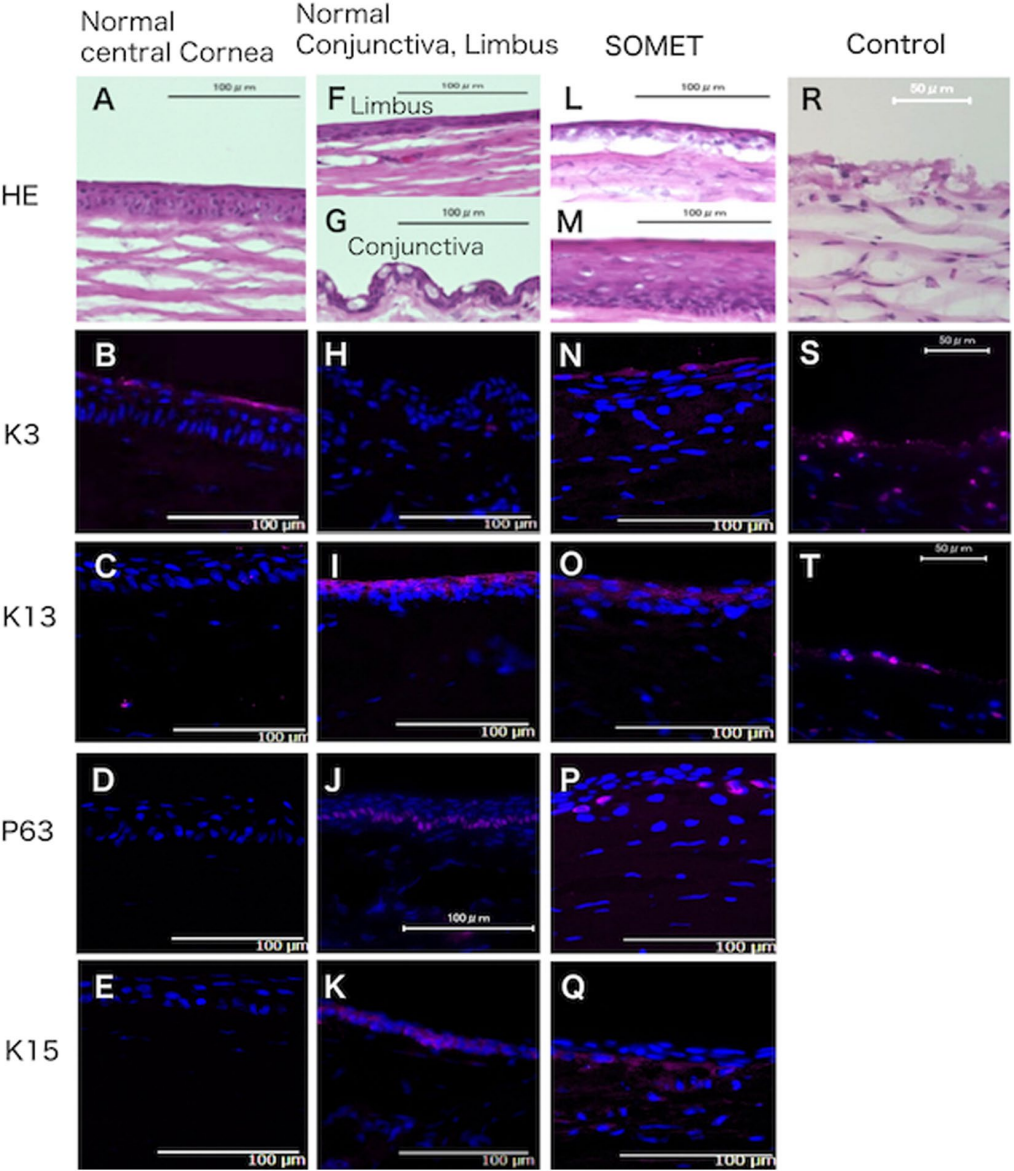
Immunohistochemistry of rabbit eyes. Frozen normal and postoperative eye specimens were used for immunohistochemical examination. The left column shows images of a normal cornea (**A–E**). (**A**) Hematoxylin-eosin (HE) staining reveals five to six layers of corneal epithelial cells in the central region. K3 is positive in the cytoplasm of cells in the surface layer of the epithelium (**B**), while K13 (**C**), p63 (**D**), and K15 (**E**) are negative in all layers. The central column shows images of a normal limbus and conjunctiva (**F–K**). HE staining of the normal limbus and conjunctiva is displayed in (**F,G**), respectively. K3 is negative in the normal conjunctiva (**H**), while K13 shows cytoplasmic positivity in the upper layer of the normal limbus (**I**). Nuclear p63 positivity is seen in the basal layer of the limbus (**J**), along with cytoplasmic K15 positivity in basal layer cells (**K**). The right column shows the central cornea of a SOMET group eye (**L–Q**). HE staining of the central cornea reveals 3–5 layers of flat epithelial cells (**L**). HE staining also shows that the transplanted oral mucosal graft consists of stratified squamous epithelium (**M**). In the deeper layer at the graft site, basal cells with a high N/C ratio exist close to the basal layer (**M**). In the expanding epithelium, the surface layer of cells demonstrates cytoplasmic K3 positivity (**N**) and K13 is also positive in the cytoplasm of superficial epithelial cells (**O**). Basal layer cells at the graft site show nuclear staining for p63 (**P**) and cytoplasmic staining for K15 (**Q**). In the Control group, HE staining revealed no epithelial proliferation and multinucleated spheres were attached to the exposed corneal stroma (**R**). Neither K3 positivity (**S**) nor K13 positivity (**T**) was detected in the Control group.

At the center of the normal rabbit cornea (Fig. 7A), K3 was positive in the surface layer (Fig. 7B), while all layers were negative for K13 (Fig. 7C), p63 (Fig. 7D), and K15 (Fig. 7E). HE-stained images of the normal rabbit corneal limbus and conjunctiva are shown in Fig. 7F,G, respectively. The conjunctival epithelium was negative for K3 (Fig. 7H), while K13 was positive in the corneal limbus (Fig. 7I) and the conjunctival epithelium (not shown). The basal layer of the corneal limbus showed nuclear p63 positivity (Fig. 7J) and cytoplasmic K15 positivity (Fig. 7K). After SOMET was performed, three to five layers of epithelial cells were revealed by HE staining (Fig. 7L). Stratified squamous epithelium was seem to have formed from the transplanted oral mucosal grafts (Fig. 7M). In epithelium expanding from the grafts, a single surface layer of epithelial cells was K3-positive, as in the normal cornea (Fig. 7N). K13 was also positive in the epithelial layer (Fig. 7O), consistent with the findings in normal rabbit oral mucosa (Fig. 4). There was no p63 or K15 staining in the expanding epithelium (not shown). However, basal cells at the graft sites were positive for p63 (Fig. 7P) and K15 (Fig. 7Q). No epithelial proliferation was noted in Control eyes and HE staining showed multinucleated spheres attached to the exposed corneal stroma (Fig. 7R). In addition, staining for K3 (Fig. 7S) and K13 (Fig. 7T) was completely negative in Control eyes.

## Discussion

This study demonstrated that total LSCD in rabbits can be completely restored by simple autografting of uncultured oral mucosal tissue onto the denuded corneal stroma. The method we used was simpler than those reported previously and dispase treatment resulted in the mucosal grafts becoming transparent more rapidly than with other techniques. In brief, the neovascularization score and fluorescein staining score were significantly lower in the SOMET group compared with the Control group, and immunohistochemistry showed that the cornea was covered with K3/K13-positive stratified epithelium at 2 weeks after transplantation in the SOMET group. Our technique for treating LSCD involved covering the grafts with a SCL and performing tarsorrhaphy for graft fixation, but we did not use graft sutures, amniotic membrane, and/or glue. Our results suggest that this simple SOMET technique could be used clinically to treat bilateral LSCD.

Because severe LSCD accompanied by symblepharon is often bilateral, attention has been focused on autologous cell sources other than the corneal limbus, and the oral mucosa has been investigated as an option for ocular surface reconstruction^23^. Direct oral mucosal transplantation without culture after dispase treatment was reported to be successful in rabbits with partial corneal limbal scraping^24^. In that study, grafts were sutured over the wounds at the peripheral corneal-limbal junction. Despite no anti-inflammatory treatment after transplantation, one eye in the allograft group (n = 13) and all four eyes in the autograft group (n = 4) showed no rejection at 120 days, suggesting good compatibility of dispase-treated oral mucosal epithelium with the ocular surface. In rats with LSCD created by alkali burns, transplantation of sutured autologous oral mucosal strips without using amniotic membrane was also reported to achieve favorable results^25^. While the graft become detached in one eye, re-epithelialization around the graft was observed from 2 to 5 days after surgery and was completed within 1 to 3 weeks in the remaining 6 eyes. This report suggested that direct transplantation of uncultured oral mucosal epithelium was effective for LSCD in rats. Clinically, grafted oral mucosa has been applied for reconstruction of the conjunctival fornix after enucleation of the eyeball and for reconstruction of the filtration bleb after glaucoma surgery^23,26^. In patients with ocular pemphigoid and severe fibrous symblepharon, sutured oral mucosa grafts were employed to reconstruct the conjunctival fornix, but most of the grafts failed within a 2-year follow-up period, presumably because fibrosis of the ocular surface was severe and preoperative/postoperative anti-inflammatory therapy was inadequate^27^. Conversely, there have been several reports of successful ocular surface reconstruction by direct transplantation of uncultured oral mucosal epithelium (attached with sutures and glue) to encircle the entire limbus in LSCD patients combined with amniotic membrane co-transplantation^28^ or temporary amniotic membrane transplantation^29^. In this study, we simplified the surgical technique and materials by omitting the use of amniotic membrane, graft sutures, and glue. We achieved ocular surface re-epithelialization and recovery of corneal transparency in the SOMET group, indicating that our simple technique was effective for LSCD. Many reports have been published about transplantation of cultured oral mucosal epithelium in animal and clinical studies^15–19,30,31^. There are three common points between our method and the techniques used in these other studies: 1) transplantation of oral mucosal epithelial stem cells, 2) reconstruction of bilateral LSCD, and 3) expression of the same proteins as native oral mucosal epithelium (CK3 and CK13) by the grafted epithelium. Transplantation of cultured oral mucosal epithelium may have the advantage of an anti-inflammatory effect because the cultured epithelial sheet covers the entire cornea and protects it after surgery. At hospitals with tissue culture facilities, transplantation of cultured oral mucosal epithelium is undoubtedly a good choice for binocular LSCD. However, our SOMET technique has the advantage of providing easy to access treatment for LSCD patients because it can be performed in hospitals where cell culture facilities and amniotic membrane are unavailable.

We treated the oral mucosal tissue with dispase before transplantation, and we used a SCL bandage and tarsorrhaphy to promote efficient attachment of undifferentiated cells in the mucosal epithelium to the denuded corneal stroma. Dispase treatment of the mucosal grafts completely removed any residual unwanted submucosal connective tissue (Fig. 1C) without loss of the basal cell markers p63 and K15 (Fig. 5), suggesting that digestion by dispase can promote attachment of oral mucosal grafts while maintaining the basal layer. Because subepithelial connective tissue is tightly adherent to the basal layer of the oral mucosal epithelium (Fig. 1C), using grafts without dispase treatment is associated with a risk of visual impairment and failure of attachment to the corneal stroma. Dispase treatment yields a pure oral mucosal epithelial graft with similar transparency to the limbal epithelium that can be transplanted directly onto the corneal stroma. The grafts we applied were milky in appearance immediately after the operation, but gradually became transparent within 1 to 2 weeks, as shown in Fig. 3 (left). These oral mucosal epithelial grafts showed a stratified squamous epithelial structure at 2 weeks after transplantation (Fig. 7M), and the cornea was completely covered by a smooth epithelial surface even where grafts had not been placed (Fig. 7L). According to previous reports about oral mucosal epithelial transplantation, grafts without dispase treatment remained cloudy for several weeks after surgery or for 3 months postoperatively in humans^23,26^. Clinical studies of SLET have also shown that limbal epithelial tissue grafts was observed for approximately 6 months postoperatively^20^. Accordingly, it can be suggested that dispase treatment of oral mucosal epithelium before grafting contributes to tight adhesion to corneal stroma and faster recovery of visual acuity compared with previous methods^20,23,26^.

In patients with corneal erosion, a SCL bandage has been reported to be effective for reducing pain and foreign body sensation, promoting epithelialization, and preventing chronic inflammation and neovascularization^32–34^. In one rabbit from the SOMET group, loss of the SCL led to graft failure and re-epithelialization was not observed at 2 weeks after surgery. Because we did not use sutures or glue, the SCL bandage is essential for stable fixation of the grafts. In animals, tarsorrhaphy is critical for maintaining the SCL *in situ*, but it is too invasive in humans. As a substitute for tarsorrhaphy, an eye patch may be effective when this procedure is applied clinically. We did not utilize amniotic membrane in our SOMET procedure. Amniotic membrane has previously been used in a wide variety of ocular surface reconstruction techniques, such as amniotic membrane transplantation^5^, limbal transplantation^10,11^, cultured corneal epithelial sheet transplantation^13^, cultured conjunctival epithelial cell sheet transplantation^14^, COMET^15–19^, and SLET^20^. However, we found that using fibrin glue with human amniotic membrane induced severe ocular surface inflammation after transplantation in rabbits (unpublished observation 2017, Inamochi A). It is unknown whether this occurred because the fibrin glue and amniotic membrane grafts were derived from humans and not rabbits, but the present results suggest that ocular surface reconstruction without amniotic membrane may be an option for LSCD.

Immunohistochemistry showed that two markers of differentiated oral mucosal epithelium, K3 and K13, were expressed in cultured epithelial sheets (Fig. 6) and in graft specimens from the SOMET group at 2 weeks after transplantation (Fig. 7N,O), consistent with other reports on transplantation of cultured oral mucosa in rabbits^30,31^. Markers of undifferentiated epithelial cells, P63^35^ and K15^36^, were present in the basal layer of graft specimens from the SOMET group (Fig. 7P,Q), whereas there were no P63- or K15-positive cells at other sites of the cornea. These findings indicate that undifferentiated oral mucosal epithelial cells were supplied by the oral mucosal grafts. On the other hand, the cultured oral mucosal epithelial sheets were only positive for p63, but not for K15 (Fig. 6). Conventional culture systems using serum and mouse 3T3 feeder cells can preserve expression of the undifferentiated epithelial cell marker K15 in oral mucosal epithelium^37–39^, but K15 expression does not persist during serum- and feeder-free culture^40,41^. In cultured corneal limbal epithelial cells, K15 expression was maintained by serum- and feeder-free culture medium containing keratinocyte growth factor, but not EGF^40^. Although modification of the culture method may be able to overcome the decrease or loss of K15 expression during culture of oral mucosal epithelium, using grafts containing K15-positive cells for SOMET without culturing epithelial sheets is simpler and could be advantageous for contributing long-term successful engraftment in patients with LSCD.

In summary, we developed a simple technique for transplantation of uncultured oral mucosal epithelium after dispase treatment, employing a SCL bandage and tarsorrhaphy to avoid the use of graft sutures, glue, or amniotic membrane. This technique was effective in a rabbit model of total LSCD. Our SOMET technique for bilateral LSCD is characterized by simplicity, no risk of rejection, and low cost. Although further investigations are required to assess the adhesion and effectiveness of grafted oral mucosal epithelium in humans, this technique could become a new option for ocular surface reconstruction in patients with bilateral LSCD.

## Materials and Methods

### Study design and animals

Eight female New Zealand white rabbits (aged 12 to 14 weeks and weighing about 2.5 kg) were assigned to a simple oral mucosal epithelium transplantation (SOMET) group (n = 4) or a Control group (n = 4). General anesthesia was induced by intramuscular injection of xylazine (5 mg/ml) and ketamine (50 mg/ml). In all rabbits, the right eye was used for this study. Evaluation was performed at 1 and 2 weeks after surgery, and ocular specimens were excised at 2 weeks postoperatively after the rabbits were killed by intracardiac injection of sodium pentobarbital (Somnopentyl, Kyoritsuseiyaku, Tokyo, Japan). Animals were treated in accordance with the animal experiment protocol approved by the Animal Experiment Committee of the University of Tokyo and the ARVO Statement for the Use of Animals Research in Ophthalmic and Vision Research.

### Harvesting of oral mucosa and separation of epithelium

After the oral cavity was disinfected with povidone iodine, the mucosa was grasped with hooked forceps and a 3 × 4 mm piece of mucosal tissue was excised with spring scissors. Separation of the oral mucosal epithelium from the submucosal tissue was compared between pulling or peeling with two pairs of forceps and treatment with 1.2 IU of dispase at 37 °C for 40 minutes, as described elsewhere^30^. The epithelium was washed with saline after dispase treatment.

### Culture of separated oral mucosal epithelial tissue

The separated oral mucosal epithelium was cultured in DMEM/Ham’s F-12 containing L-glutamine, Phenol Red, HEPES, and sodium pyruvate (Wako, Osaka, Japan) supplemented with B27, EGF and penicillin-streptomycin-amphotericin b Suspension (Wako, Osaka, Japan) in a CO_2_ incubator at 37 °C for 3 weeks. The medium was exchanged after the first week, every other day during the second week, and every day during the third week. In order to promote tissue adhesion, sufficient medium was added to completely cover the surface of the mucosal tissue for the first week.

### Surgical technique

**Figure 8:**
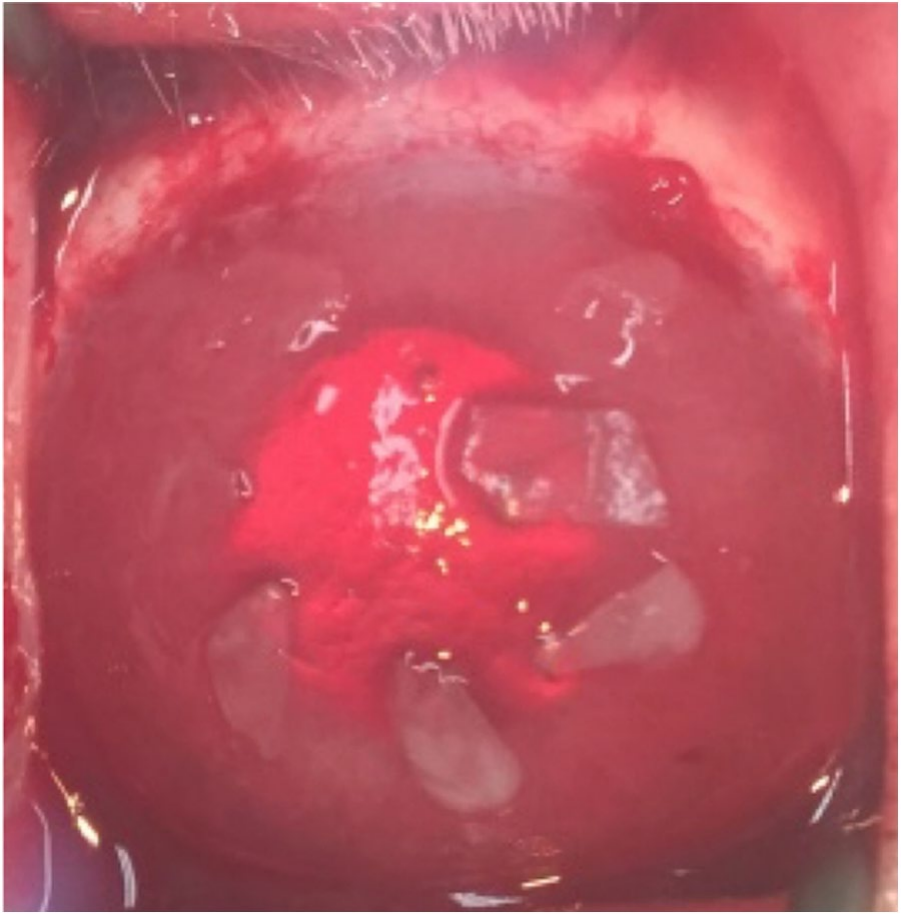
Anterior segment of an eye in the SOMET group immediately after placement of the grafts. The grafts of oral mucosal epithelium were washed with saline and arranged on the denuded corneal stroma (not around the limbus or bare sclera) with the epithelial surface upward. Grafts were placed without any glue or sutures.

To create a rabbit model of LSCD, the conjunctiva and subconjunctival tissue were excised to 5 mm outside the limbus and the corneal epithelium including the limbus was completely removed for 360 degrees in one eye under anesthesia. This procedure was done in 8 animals to create LSCD. In the SOMET group (n = 4), the harvested oral mucosal epithelium was cut into 8 to 10 pieces with scissors and treated with dispase, as described above. After washing with saline, pieces of the treated epithelium were arranged on the denuded corneal stroma with the epithelial surface upward (Fig. 8). These oral mucosal epithelial grafts were placed without any glue or sutures. In the Control group (n = 4), transplantation of grafts onto the keratectomized cornea was not done. In all animals, a SCL was placed over the eye after application of ointment containing betamethasone sodium phosphate and fradiomycin sulfate (Rinderon – A ointment, Shionogi, Osaka, Japan). Tarsorrhaphy was done with 2 to 3 vertical stitches to keep the SCL in position. Ointment was applied once a day for the first week after surgery and then every other day during the second week.

### Clinical scores

Slit lamp examination was conducted at 1 and 2 weeks after surgery to score corneal neovascularization and epithelialization. Neovascularization was scored as follows: Grade 0, no neovascularization of the cornea (score = 0); Grade 1, neovascularization ≤2 mm (1); and Grade 2, neovascularization >2 mm (score = 2). Epithelialization was scored as follows: Grade 0, no fluorescein staining of the cornea (score = 0); Grade 1, staining <1/4 (score = 1); Grade 2, staining from 1/4 to 1/2 (score = 2); and Grade 3, staining >1/2 (score = 3). If the SCL fell off before follow-up examination, the eye was excluded from evaluation. Scores were analyzed by the Mann-Whitney U test. Following evaluation at 2 weeks after surgery, all treated eyes were excised and frozen blocks were prepared for immunohistochemical examination.

### Immunohistochemistry

Samples were fixed in 4% paraformaldehyde. Paraffin blocks of cultured cell sheets and frozen blocks of the ocular specimens were prepared. The frozen blocks were cut into 5 μm sections on a cryostat, while the paraffin blocks were cut into 4 μm sections. Then the sections were washed with phosphate-buffered saline (PBS) and blocked with 10% goat serum / PBS for 30 minutes at room temperature. The primary antibodies were K3 (x1000, [AE5] ab77869; Abcam, Cambridge, MA), K13 (x200, [AE8]; Santa Cruz, Dallas, Texas), K15 (x100, [LHK15] sc-47697; Santa Cruz, Dallas, Texas), and p63 (x50, [4A4] ab735: Abcam, Cambridge, MA). Each antibody was diluted with 0.1% bovine serum albumin (BSA) / PBS and added to the sections for overnight incubation at 4 °C, followed by three 5-min washes with PBS, incubation for 90 minutes at room temperature with the goat anti-mouse IgG (H + L) cross-adsorbed secondary antibody, Alexa Fluor 647 (x200; Thermo Fisher Scientific, Waltham, MA), and three further 5-min washes. Subsequently, the sections were mounted in medium with 4′,6-diamidino-2-phenylindole (DAPI).

### Statistical analysis

Results are expressed as the mean ± standard deviation. The Mann-Whitney U test was used to compare the two groups and *P* < 0.05 was considered to indicate statistical significance. All analyses were carried out by using JMP® Pro 14.1.0 software.
